# Current approaches to handling rescue medication in asthma and eczema randomized controlled trials are inadequate: a systematic review

**DOI:** 10.1016/j.jclinepi.2020.05.027

**Published:** 2020-09

**Authors:** Anca Maria Chis Ster, Victoria Cornelius, Suzie Cro

**Affiliations:** Imperial College London, Imperial College Clinical Trials Unit, 1st Floor Stadium House, 68 Wood Lane, London W12 7RH, UK

**Keywords:** Rescue medication, Randomized controlled trials, Statistical methods, Reporting

## Abstract

**Objectives:**

The objective of this study was to examine how rescue medication is defined, reported, and accounted for in randomized controlled trials (RCTs) in eczema and asthma populations.

**Study Design and Setting:**

This is a systematic review of phase II/III RCTs evaluating monoclonal antibodies for treating chronic eczema or asthma. A search of EMBASE, MEDLINE, and the Cochrane Central Register of Controlled Trials was conducted to identify eligible RCTs.

**Results:**

Sixty published RCTs were identified, of which 60 (100%) allowed use of rescue medication but only 28 (47%) reported its use. Twenty-seven (45%) articles summarized rescue use by arm, with an average of 25% (95% CI (17%, 36%)) greater use in the placebo arm. Nine (15%) trials undertook an analysis that adjusted the primary treatment effect estimate for rescue medication use, but 8 of these used a suboptimal approach using single imputation, including 4 which used “last observation carried forward” after setting postrescue data to missing.

**Conclusion:**

Rescue medication use in eczema and asthma trials evaluating monoclonal antibodies is often permitted, but not routinely reported. There is evidence of imbalance in rescue use between arms, but few articles attempted to estimate a rescue-adjusted treatment effect. In trials that did, the methods used were suboptimal which could introduce bias.

**What is new?**

**Key findings**
• Rescue medication in chronic eczema and asthma trials is often permitted, but not routinely reported. Few trials attempted to account for rescue medication use in the statistical analysis even though rescue use was on average greater in the placebo arm. Trials that did aim to obtain a rescue-adjusted treatment effect frequently used methods that are highly vulnerable to introducing bias into the estimate.

**What this adds to what was known?**
• This review demonstrates that, while rescue medication is frequently permitted in trials alongside the treatment under evaluation, there is minimal attention or interest in isolating the underlying treatment effect of interest. Obtaining an unbiased treatment effect estimate adjusted for postrandomization variables, such as the use of rescue medication, requires statistical approaches that have been emerging over the last 10 years, and this review demonstrates that these approaches are currently not being used.

**What is the implication and what should change now?**
• Reporting of rescue medication by arm should be encouraged in published articles. Guidance on statistical approaches that can provide unbiased adjustments for rescue medication use could help encourage investigators to present alternative treatment effects relevant to the patients and prescribers and support better statistical practice in randomized controlled trials.

## 1 Introduction

Randomized controlled trials (RCTs) are the gold standard for determining efficacy and effectiveness of new drug therapies. When participants experience an inadequate therapeutic effect or an exacerbation of symptoms, they may start nontrial treatment (which we throughout refer to as rescue medication) in addition to their randomized treatment in the trial [1]. Rescue medication presents a problem across the board; it is particularly present in chronic conditions, such as asthma and eczema, but also in areas such as mental health, cardiovascular health, and diabetes [2]. Access to rescue medication is often permitted for ethical reasons and to encourage recruitment and retention of participants. Typically in trials an intention-to-treat (ITT) analysis will be performed, whereby all patients are analyzed as randomized regardless of any postrandomization events such as rescue medication use [3]. However, allowing participants access to rescue medication in addition to the study drug treatment may impact the treatment effect estimate; typically by reducing the effect [1]. The magnitude of the impact partly depends on the distribution of the use of rescue medication and will be of greater consequence when an unequal proportion of participants per arm receive rescue medication, which may be expected in a controlled therapeutic trial including a placebo arm. An ITT analysis that does not adjust for rescue medication provides an estimate of the treatment effect following the treatment policy strategy. This is a pragmatic approach to compare treatment groups [4] but explains only the effect of the assigned treatment [5].

It is often of value to obtain a rescue-adjusted treatment effect which provides a measure of how well the randomized treatment works in isolation, which may be of more interest to patients [6]. It has previously been discussed that entering postrandomization variables (such as rescue medication) as covariates in the analysis is not a suitable method of adjustment [1,7]. Some guidance on statistical methods to take account of rescue medication use has been reported; rank-based methods, responder analysis, multilevel regression–based methods using time-varying covariate adjustment, and methods of analysis which set postrescued data missing and assume missing at random (MAR), such as multiple imputation and multilevel models, have all been explored as methods to account for rescue medication [1,2]. Recently, the estimation of alternative treatment effects that account for the occurrence of intercurrent events in trials, such as rescue medication use, has been brought to the forefront by the publication of an addendum to the International Standard drug trial Guidelines (ICHE9(R1), 2017 [6]) on estimands and sensitivity analysis in clinical trials. Within these guidelines, alternative analysis strategies in the presence of rescue medication are outlined. Examples of such strategies include integrating the use of rescue therapy into the outcome of interest (i.e., composite strategy), estimating the treatment effect if rescue medication had not been available (i.e., hypothetical strategy), estimating the treatment effect for the strata of individuals who would not take rescue therapy (i.e., principle stratum strategy), or estimating the treatment effect while on treatment conditions only (while on treatment strategy). However, the guidance does not provide details on statistical methods for estimating such effects and there is currently no consensus on what are the most suitable approaches to obtain rescue-adjusted treatment estimates.

This is the first systematic review we are aware of aiming to identify statistical approaches used to adjust for rescue medication in the context of RCTs in chronic disease conditions. We were particularly interested to discover how rescue medication is described and reported in published RCTs of chronic disease and to identify the statistical approaches currently being used to adjust for the impact of rescue medication on the treatment effect estimate. As a case example, we selected RCTs evaluating a set of monoclonal antibodies for the treatment of chronic eczema or chronic asthma. This clinical application was chosen to include studies highly likely to permit rescue medication and involve trialists who were likely to be well versed in the challenges around use of rescue medication in RCTs.

### 1.1 Objectives

The main aim of this study was to evaluate current practice to adjust for rescue medication use in asthma and eczema RCTs evaluating a set of monoclonal antibodies. Our specific objectives were to estimate the proportion of RCTs that define and report the use of rescue medication, examine how rescue medication use is summarized and evaluate methods used to adjust for the impact of rescue medication on the primary outcome. The protocol is registered on the International Prospective Register of Systematic Reviews [8].

## 2 Methods

### 2.1 Literature search

We searched for articles in EMBASE, MEDLINE, and the Cochrane Central Register of Controlled Trials (CENTRAL) libraries with no restriction on the publication date. Search strategies were developed using database-specific indexing terms and keyword searching in titles and abstracts to fit the following structure; [randomised controlled trials] AND [asthma OR eczema] AND [clinical and brand name for each intervention]. Keyword search terms to identify randomized controlled trials included terms such as “random$” or “placebo$” and we excluded articles that used terms such as “review” or “meta-analysis”. Asthma and eczema were searched using a number of alternative terms such as “asthma”, “eczema”, “atopic dermatitis”, and database-specific indexing terms. The full search strategy for each database searched is presented in Appendix 1.

### 2.2 Trial inclusion criteria

Eligible articles were phase II/III RCTs that evaluated one of the following drug interventions: benralizumab, dupilumab, mepolizumab, omalizumab, palivizumab, or reslizumab and allowed for any comparator (placebo, standard care, or other active ingredients). Trial participants had a diagnosis in either chronic eczema or chronic asthma with no restriction on age, gender, or ethnicity. The main exclusion criteria included research letters, conference abstracts, pilot studies, feasibility studies, review articles, meta-analyses, secondary analyses, subgroup analyses, and results of interim analyses. In addition, foreign literature where a translation in English was not available and trials where the primary outcome was a nonclinical outcome, that is, laboratory test results and in vivo studies.

### 2.3 Selection of trials for inclusion in the review

Search results from all libraries were exported to EndNote and duplicates were removed. Titles and abstracts were screened by one reviewer (A.C.) using the predefined eligibility criteria as specified in section 2.2. Articles were classed as ineligible if keywords, such as “systematic”, “pilot”, “meta”, “review”, “phase 1”, and “secondary” were included in the title of the article. A random 10% sample were also selected to be title and abstract screened by a second reviewer (S.C.) to confirm eligibility. The full texts of the remaining articles were then obtained and eligibility was confirmed or articles were excluded after full-text assessment.

### 2.4 Data extraction

A standardized data extraction form was developed to collect trial characteristics, such as publication year, study design, and primary population. Data extracted to address the aims of this review included the definition of rescue medication, its reported use, and statistical approach used to adjust for rescue use. All information was extracted from the primary article and online supplementary material (e.g., protocols, appendices). A single attempt was made to contact the corresponding authors of articles, where the protocol was not available online. Whole information on analysis methods used to obtain a rescue-adjusted treatment effect was extracted by two authors independently (A.C. and S.C.). Where multiple primary outcomes were specified, the outcome that was used in the sample size calculation was taken; if not available then the outcome that was specified first in the text was used. Articles that conducted several identical independent RCTs were extracted once for methodology, but separate trial results were extracted and summarized. This meant that the outcomes related to the reporting and analysis methods could be independent of one another. The full data extraction form is presented in Appendix 2.

### 2.5 Outcomes

The main outcomes were to estimate the proportion of all eligible articles that defined and reported the use of rescue medication, evaluate the statistical methods used to adjust for rescue medication use on the primary outcome, and calculate the changes in treatment effect estimates after adjustment for rescue medication. In this review, rescue medication use was any permitted medicine (except the active drug in the trial) that participants used for the relief of symptoms or acute exacerbations as defined by the trialists. To ascertain the definition provided in each article, we first searched for terms such as “rescue” or “concomitant” in the article or for well-recognized rescue medication alongside treatment. In both asthma and eczema trials, rescue medication is well recognized and well defined. For example, in chronic asthma trials, participants may be permitted to use corticosteroids, short-acting beta agonists or albuterol as a form of rescue medication.

### 2.6 Statistical analysis

Analysis was primarily descriptive. Frequencies and percentages were used to describe categorical data, whereas means, medians, standard deviations, and interquartile ranges were used to summarize continuous data. Where trials conducted a formal assessment for imbalance in rescue use between groups, their imbalance results (as *P*-value thresholds of ≤0.05 and >0.05) were compared against the presence of a rescue-adjusted analysis for the primary outcome. The impact of adjusting for use of rescue medication was quantified by calculating the percentage change between treatment estimates with and without adjustment for rescue medication, alongside changes in the *P*-values. Binary outcomes were pooled using a random effects model and the method of DerSimonian and Laird, combining all active arms to form one arm in multiarm studies [9].

## 3 Results

### 3.1 Results of the search

**Fig. 1:**
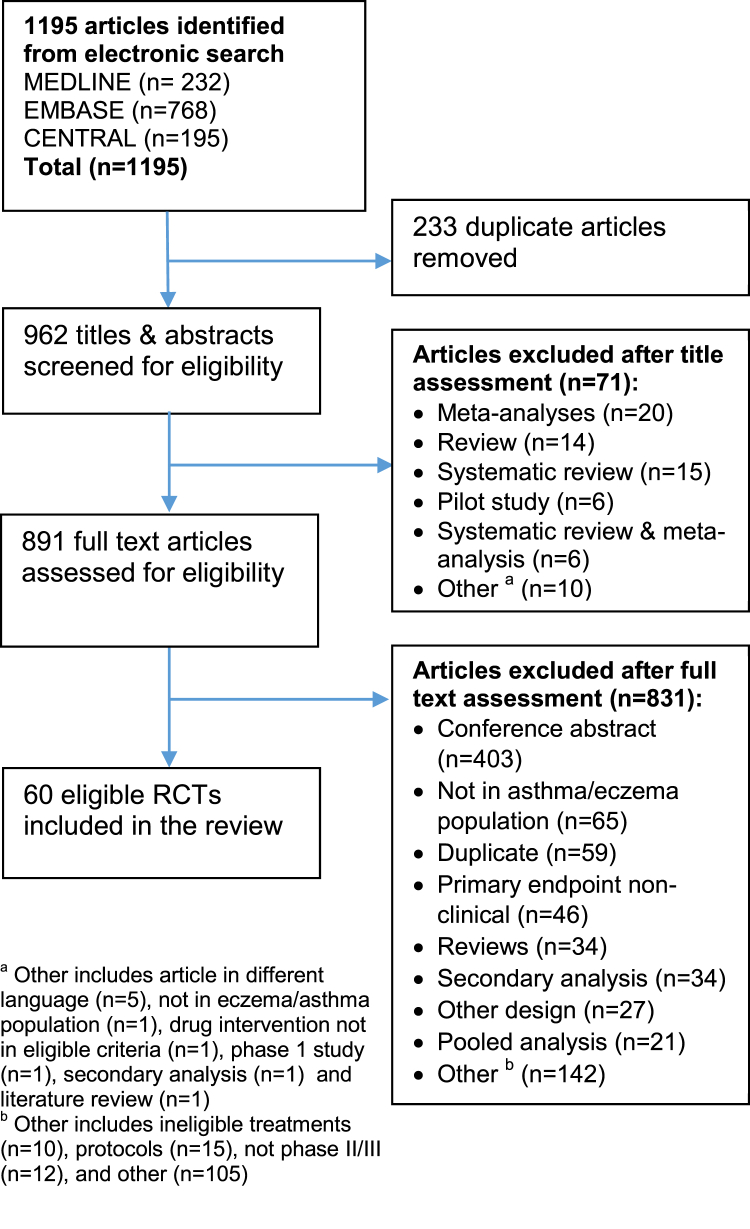
Flow diagram of trial selection.

The flow diagram of the trial selection process is shown in Fig. 1. We identified 1,195 articles through database searching: 768 results from EMBASE, 232 results from MEDLINE, and 195 results from the CENTRAL library. There were 962 unique articles to screen after the removal of 233 duplicate articles. A random sample of 96 (10%) articles was double-screened for eligibility. The double screening was predominately based on abstracts and titles, but the second reviewer also referred to the full texts of the articles where eligibility was not clear from the abstract or title alone. There was 100% agreement between two reviewers (A.C. and S.C.). Any further subsequent uncertainties on eligibility for the remaining articles in full-text review were double-screened in an ongoing review. Overall, 162 (16.8%) article were double-checked for eligibility. Seventy one articles were found to be ineligible based on keyword search (listed in section 2.1) in titles, and a further 832 records were ineligible with the most common reason being conference abstracts (*n* = 403). Sixty eligible articles were included in the review.

### 3.2 Characteristics of included trials

Characteristics of the 60 included trials are summarized in Table 1. Most articles were on asthmatic participants, 52 (87%), and the most common intervention evaluated was omalizumab, 27 (45%). Most of the trials had a parallel-arm design (98%).

**Table 1:** Characteristics of the RCTs in the review

Diagnosis of participants	*N* (%)
Asthma	52 (87)
Eczema	8 (13)
Drug interventions	
Omalizumab	27 (45)
Dupilumab	12 (20)
Mepolizumab	11 (18)
Benralizumab	6 (10)
Reslizumab	4 (7)
Severity of disease population a	
Severe	17 (28)
Moderate – severe	28 (47)
Mild – moderate	8 (13)
Any	7 (12)
Study design	
Parallel arm	59 (98)
Crossover	1 (2)
Number of parallel active treatment arms	
1	43 (72)
2	13 (22)
3	2 (3)
4	1 (2)
5	1 (2)
Age range of population	
Adults, all ≥ 18	56 (93)
Pediatric, all ≤ 12	2 (3)
Pediatric, adolescents and adults	2 (3)
Protocol available	
Yes	13 (22)
No	
Requested	
No response	8 (13)
Reply—confidential	3 (5)
Not requested	36 (60)
Mean (SD)	Median (IQR)
Number of patients randomized	
346.0 (355.6)	258.5 (57.5–535.5)
Treatment duration	
25.9 (16.4)	22 (15–32)

*Abbreviations:* SD, standard deviation; IQR, interquartile range; RCT, randomized controlled trials.

a Stated in article or determined by physiologic measures of lung function [10].

### 3.3 Outcomes of the trials

We ascertained that all 60 (100%) trials permitted participants to use rescue medication throughout the trial, 58 (97%) clearly stated this in the article and/or supplementary material (see Table 2 for outcomes on rescue medication use). This meant that keywords such as “rescue” or “concomitant” or any well-recognized rescue medication did not appear in 2 (3%) of the results articles in this review.

**Table 2:** Outcomes of rescue medication use

Outcomes on rescue medication use	*N* (%)
Was rescue medication allowed in the trial?	
Yes	60 (100)
Did the article or supplementary material clearly report whether rescue medication was permitted?	
Yes	58 (97)
No	2 (3)
Were some data on rescue medication use reported in the article?	
Yes	28 (47)
No	32 (53)
Were summary statistics provided on the use of rescue medication by arm^a^?	
Yes	27 (45)
No—Used a *P*-value to describe statistical difference in the number of participants on rescue medication	1 (2)
No information on rescue medication use reported	32 (53)
How was rescue medication use summarized?	
Change from the baseline in the average number of uses per individuals per time period (e.g., weekly/daily)^a^	15 (25)
Proportion of participants who received rescue a	6^b^ (10)
Average number of uses per individual per time period (e.g., weekly/daily) a	4 (7)
Total number of uses per individual over study period a	1 (2)
Total number of uses over study period a	1 (2)
Proportion of rescue free days a	1^b^ (2)
Described no significant difference in the number of participants on rescue medication	1 (2)
No information on rescue medication use reported	32 (53)
Tested for statistical imbalance in rescue medication use between arms	
Yes	20 (33)
No	40 (67)
Was rescue medication adjusted for in an analysis? c	
Yes	9 (15)
No	51 (85)

a Numerical Data presented by arm or between arm differences presented.

b One trial reported two summaries on rescue medication use.

c Either primary or sensitivity analysis.

Only 28 (47%) articles reported some form of data on rescue medication use. For example, one article used a *P*-value to describe statistical significance in the number of participants on rescue medication, but no summary statistics were presented. Twenty-seven (45%) articles summarized rescue use quantitatively by arm, or the difference in use between arms, in some way. The most frequently used summary of rescue medication use (*n* = 15, 25%) was the change from the baseline in the average number of uses per individual per time point (e.g., weekly/daily), followed by the proportion of patients using rescue medication (*n* = 6, 10%, see Table 2). Pooled data (*n* = 6) found that the proportion of patients using rescue medication was on average 26% (95% CI (17%, 36%)) greater in the placebo arm. Although it was inappropriate to combine any other rescue use statistics in this pooled analysis, it was clear from the remaining results that rescue use was greater in the placebo arm, regardless of the summary measure used.

A total of 20 (34%) articles conducted a formal assessment to test for any statistical imbalance in rescue medication use between arms. Traditionally, an imbalance may be identified by testing at the 5% significance level. Results of the presented imbalance tests (*P* ≤ 0.05, *P* > 0.05) were assessed by whether trialists performed a rescue-adjusted analysis for the primary outcome and are presented in Appendix 3. Of the 20 articles that tested for an imbalance in rescue medication use between arms, 12 (60%) found some evidence (*P* < 0.05), but only 2 (17%) of these articles adjusted for rescue in their analysis.

We found 9 (15%) articles that included a rescue-adjusted analysis of the primary outcome. While the trials included in this review were predominantly from asthma populations (87%), we found that rescue medication was taken into account in the analysis of 7 of 8 (88%) of the remaining eczema trials in comparison with 2 of 52 (4%) asthma studies. Methods of adjustment included setting postrescue data to missing or categorizing participants who have been subject to rescue medication as nonresponders (e.g., in a binary outcome setting, this means the participant has not achieved the primary endpoint). Of these, 8 (89%) of these 9 trials used a suboptimal approach; half used last observation carried forward (LOCF) after setting postrescue data to missing (4/9, 44%) and the other half treated participants who took rescue as nonresponders (4/9, 44%). All rescue-adjusted methods of analysis are displayed in Table 3.

**Table 3:** Method of rescue-adjusted analysis

Primary method of analysis	*N* (%)
Analysis of covariance (ANCOVA) model with efficacy data set to missing after rescue medication use (or dropout) and LOCF method used to impute missing values	4 (44)
Cochran-Mantel-Haenszel (CMH) test stratified by randomization strata with participants specified as being nonresponders at rescue medication initiation (or study withdrawal)	4 (44)
Mixed model with assessments excluded from the full analysis set (FAS) if they were obtained at scheduled visits that were preceded by a limited subset of medications that could confound interpretation	1 (11)
Total	9 (100)

*Abbreviation:* LOCF, last observation carried forward.

All 9 rescue-adjusted analyses were the primary analysis of the study with 4/9 (44%) presenting an additional sensitivity analysis that did not adjust for rescue medication. Unadjusted trial analyses included all observed data in their analysis regardless of rescue medication use and were analyzed using the same analysis set as the primary analysis (ITT). One article had reported 2 RCTs with the same design/methods and these results were individually extracted and summarized. All raw data from articles that presented both adjusted and unadjusted analyses are presented in Table 4. Odds ratios from binary outcome studies are presented and pooled in Appendix 4. We also investigated whether the year the article was published (grouped in tertiles by 1997–2010, 2011–2014, and 2015–2019) was associated with whether or not a rescue-adjusted analyses had been undertaken and found strong evidence (*P* = 0.006) that the prevalence of a rescue-adjusted analysis was greater among more recently published articles. The proportion of articles that are accounted for rescue medication for each year group described previously was 0%, 11%, and 33%, respectively.

**Table 4:** Effect of eczema and asthma treatment on various outcomes, adjusted and not adjusted for rescue medication

Binary outcomes											
		Rescue-adjusted analysis				Non–rescue-adjusted analysis				Rescue-adjusted vs. non–rescue-adjusted	
Study	Active drug	Placebo *n*/*N* (%)	Active drug *n*/*N* (%)	Treatment effect^a^ (% difference)	*P*-value	Placebo *n*/*N* (%)	Active *n*/*N* (%)	Treatment effect^a^ (% difference)	*P*-value	Difference in treatment effect %^a^	*P*-value (% change)
Blauvelt et al., 2017 [11]	Dup Q2W	39/315 (12.4)	41/106 (38.7)	26	<0.001	49/315 (15.6)	41/106 (38.7)	23	<0.001	3.2	<0.01
	Dup QW		125/319 (39.2)	27	<0.001		134/319 (42.0)	27	<0.001	0.4	<0.01
de Bruin-Weller et al., 2018 [12]	Dup Q2W	32/108 (29.6)	67/107 (62.6)	33	<0.001	35/108 (32.4)	69/107 (64.5)	32	<0.001	0.9	<0.01
	Dup QW		65/110 (59.1)	30	<0.001		67/110 (60.9)	29	<0.001	1.0	<0.01
Simpson et al., 2016 [13] d	Dup Q2W	23/224 (10.3)	85/224 (37.9)	28	<0.001	29/224 (12.9)	91/224 (40.6)	28	<0.001	0.0	<0.01
	Dup QW		83/223 (37.2)	27	<0.001		85/223 (38.1)	25	<0.001	1.8	<0.01
Simpson et al., 2016 [13] d	Dup Q2W	20/236 (8.5)	84/233 (36.1)	28	<0.001	25/236 (10.6)	87/233 (37.3)	27	<0.001	0.8	<0.01
	Dup QW		87/239 (36.4)	28	<0.001		91/239 (38.1)	28	<0.001	0.4	<0.01
Pooled binary treatment effect (95% CI)		114/883	637/1,561	28 (24, 31)	<0.001	138/883	665/1,561	27 (23, 30)	<0.001		
Continuous outcomes^c^											
Study	Active drug	Rescue-adjusted analysis				Non–rescue-adjusted analysis				Rescue-adjusted vs. non–rescue-adjusted	
		Placebo *N* LSM (SE)	Active drug *N* LSM (SE)	LSM Δ^b^ (95% CI)	*P*-value	Placebo *N* LSM (SE)	Active drug *N* LSM (SE)	LSM Δ^b^ (95% CI)	*P*-value	Difference in LSM Δ^a^ (LSM Δ % change)	*P*-value (% change)
Bjermer et al., 2016 [14]	Reslizumab 0.3 mg	103 0.126 (0.0549)	101 0.242 (0.056)	0.115 (0.02, 0.22)	0.024	103 0.127 (0.0547)	101 0.238 (0.0553)	0.111 (0.012, 0.211)	0.028	0.004 (3.6)	−0.163
	Reslizumab 3.0 mg		102 0.286 (0.055)	0.16 (0.06, 0.26)	0.002		102 0.286 (0.0546)	0.159 (0.060, 0.258)	0.002	0.001 (0.6)	0.000

N.B., All primary outcomes were positive—that is, an increase in the primary outcome indicated an improvement in the observed condition.

a Treatment effect = active—placebo.

b Treatment difference = reslizumab—placebo.

c As per protocol, continuous outcomes were planned to be converted to ORs using Hasselblad and Hedges' method but this was not achieved because study reported LSM from adjusted regression model.

d One article reported 2 identical RCTs.

Rescue-adjusted vs. non–rescue-adjusted (active vs. placebo) treatment effect differences were all non-negative suggesting a greater treatment difference between groups in a rescue-adjusted analysis. The treatment effect was large across all trials, indicating that the monoclonal antibody drugs were highly effective in improving disease symptoms. There were no notable differences between *P*-values in rescue-adjusted analyses vs. non–rescue-adjusted analyses implying there is no evidence of important clinical differences between the adjusted and unadjusted analyses. All five RCTs presented in Table 4 display highly significant *P*-values in both rescue-adjusted and non–rescue-adjusted analyses.

The pooled binary treatment effect for the rescue-adjusted analysis was 28% (95% CI (24%, 31%)) in comparison with 27% (95% CI (23%, 30%)) for the non–rescue-adjusted analysis. On the OR scale, this result corresponds to a 16% increase in the odds of achieving the primary outcome in the rescue-adjusted analysis (4.79, 95% CI (3.82, 6.02)) than in the non–rescue-adjusted analysis (4.13, 95% CI (3.34, 5.11)). Pooled binary treatment effects are displayed in Appendix 4.

## 4 Discussion

Our findings suggest that rescue medication use is not commonly reported in trials of asthma and eczema where it is permitted and it is less commonly attempted to be adjusted for in a supplementary or primary analysis. Previous research has discussed that the treatment effect estimate will not meaningfully change in a rescue-adjusted analysis unless there is some disparity in the use of rescue medication between randomized arms [1]. It is therefore important that the use of rescue medication is reported by randomization arm along with the time pattern of use [1]. However, this review found that less than half of trials reported data on rescue medication use and even less had assessed the imbalance in rescue use between arms.

Despite some evidence of imbalance between arms, little took action to adjust for rescue medication in their analysis and those that did adjust for rescue medication often used methods that were subject to bias. Although we acknowledge that it may be of interest for trialists to publish evidence for an imbalance in rescue medication to support a more favorable conclusion in a therapeutic trial, it was not possible to evaluate this bias in this systematic review. While in this review we used a *P*-value of 0.05 and below to identify statistical imbalance between arms to follow the original trials approach, we believe this is a too high bar for judgment. For future practice, we recommend trial investigators take careful consideration over the *P*-value threshold used. We suggest that a higher threshold-value would be more suitable (e.g., 0.1 or 0.2), or as an alternative we suggest evaluation of the point estimate describing rescue use in each treatment arm be used to steer away from the temptation to use the typically accepted *P*-value threshold of 0.05.

This review found that the most common strategies used to obtain rescue-adjusted treatment effect estimates were a composite analysis strategy or a hypothetical analysis strategy. The hypothetical strategy adjusted for rescue medication by setting postrescue data to equal the value at the participants' last observed time point prerescue medication initiation (LOCF analysis). This approach assumes no further decline for the rescued and will most likely achieve anticonservative estimates [15]. Participants who initiate rescue medication are more likely to have had worse clinical outcomes and continued to clinically deteriorate in the absence of rescue medication. Official guidelines have previously reported that LOCF is not appropriate under certain restrictive circumstances; namely when the patient's condition is expected to worsen [15]. Such methods have also been used to account for rescue medication in other chronic disease areas such as diabetes, despite the lack of statistical justification for its validity [16]. Simulation studies show that the last prerescue observation carried forward approach leads to biased estimates that are in favor of the active treatment group [2]. The composite strategy identified in this review adjusted for rescue medication by specifying participants as nonresponders at rescue medication initiation (or study withdrawal). Although these approaches claim to ensure that the effect of rescue medication use on the trial findings is minimized, the evidence suggests that these findings are subject to bias as participants who initiate rescue medication in a trial are more likely to have experienced considerable clinical deterioration [17].

One analysis utilized only on-treatment data in the analysis. This article used a mixed model that excluded assessments from the full analysis set if they were obtained at visits that were preceded by a limited subset of medication (including rescue medication) [14]. The excluded data were assumed MAR. This trial also imputed (using MI) for their secondary rescue-adjusted analysis aiming to test the robustness to missing data of the primary model for repeated measures analysis. The imputed model did not provide statistically different results. This result is unsurprising given MI also assumes MAR for the excluded data; however, this article did not provide details on the variables included in the imputation model so it is unclear as whether the exact same MAR assumption was made in both analyses.

There is scant literature discussing and examining methods to elicit rescue-adjusted treatment effects from RCTs. The problem is analogous to adjustment for any postrandomization variable and could be handled with a variety of approaches. Valid approaches discussed in the literature to date, specifically within the context of adjusting for rescue medication use include analyses that target a hypothetical estimand and set postrescue data missing and allow for valid inference under the missing at random assumption such as multiple imputation, or multilevel modeling [2]. An alternative composite analysis strategy that could also be used would be to use rank-based methods that assign patients on rescue medication a rank worse than the nonrescued at the endpoint of interest [1,2]. Multilevel regression based methods where rescue medication is adjusted for using a time-varying covariate have also been proposed for use; however, these rely on strong assumptions [1,2,17]. Two of the included authors of this study have recently assessed the impact of rescue medication in an RCT of omalizumab in the management of severe pediatric atopic eczema using a statistically principled missing not at random (MNAR) multiple imputation approach [18]. Data collected after the initiation of rescue medication was set as missing and a pattern-mixture multiple imputation approach [19] was used to investigate the robustness of the primary analysis results under various contextually relevant MNAR assumptions for the postrescued data [20,21]. For example, missing data for the rescued was imputed assuming a higher mean outcome (representing a worse mean response) than for the observed nonrescued, by incorporating a prespecified numerical adjustment within the imputation process. Such an approach targets a hypothetical estimand, assuming a specified change in response for the rescued in the absence of rescue medication, relative to the observed nonrescued individuals. Naturally when participants have received rescue therapy in a trial the most appropriate statistical method of analysis will depend on the research question, thus the precise estimand of interest.

Within this review, we focused on asthma and eczema trials and found a greater proportion of eczema studies conducted an analysis that accounted for rescue medication use. Different statistical practice may be occurring across these two medical areas; however, the overall numbers of eczema studies included was low. Nonetheless, the overall lack of use of more principled statistical methods identified in this review and limited discussion found in published research on this topic suggests the RCT community may benefit from targeted recommendations and explanations specific to rescue medication. There does, however, seem to be an encouraging increasing trend between the number of trialists accounting for rescue medication use in the statistical analysis and the year the article was published, and we hope this is indicative of a change in practice. However, the applied methods for adjusting for rescue medication remain inadequate and guidance on available accessible statistical approaches to adjust for postrandomization variables that can be used for rescue medication use is currently insufficient. Better guidance in this area is necessary to encourage better practice.

There is little literature that investigates the impact of the use of rescue medication in an RCT setting. The available literature focusses primarily on evaluating proposed methods using simulation studies and case studies. To our knowledge, this is the first systematic review to investigate the impact of the use of rescue medication in an RCT setting. In this specific example, we found that rescue medication use in eczema and asthma RCTs evaluating benralizumab, dupilumab, mepolizumab, omalizumab, palivizumab, or reslizumab did not have a statistical impact which altered conclusions regarding the primary treatment effect. The impact of rescue medication use will vary and will in part depend on the efficacy of the drug intervention under evaluation, the type of rescue medication and the statistical analysis undertaken. As the treatments were found to be highly effective and rescue medication less influential by comparison, any estimated treatment effect differences due to the use of sup-optimal methods cannot be well demonstrated by the cases in this review. While we did not find any statistical differences between rescue-adjusted and rescue-unadjusted treatment effects, within this review we considered a broad definition for rescue medication, defined as any permitted medicine (except the active drug in the trial) that participants used for the relief of symptoms or acute exacerbations as defined by the trialists. We acknowledge that different types of rescue medication, which may depend on factors such as disease area, disease severity, and age group administered to, may impact the treatment effect estimate differently. There was also one article that reported two RCTs. The inclusion of these trials in one article could mean that they had similar approaches to the analysis and reporting of rescue medication and may not be fully independent of one another.

While this study aimed to evaluate a unified approach to accounting for rescue medication in trials, an interesting area for future research would be to consider the impact of allowing for different types of rescue medications, varied treatment effect size, and a broader clinical and intervention context. In trials where new treatments are expected to have a more moderate impact on outcome, adjusting for rescue medication may be of greater value.

## CRediT authorship contribution statement

**Anca Maria Chis Ster:** Methodology, Software, Formal analysis, Investigation, Data curation, Writing - original draft, Writing - review & editing, Visualization. **Victoria Cornelius:** Methodology, Writing - review & editing, Supervision, Project administration, Funding acquisition. **Suzie Cro:** Conceptualization, Methodology, Validation, Investigation, Writing - review & editing, Supervision, Project administration, Funding acquisition.

## Supplementary data

Data Profile
