# Supplementary material for: Current approaches to handling rescue medication in asthma and eczema randomized controlled trials are inadequate: a systematic review
Source: J Clin Epidemiol. 2020 Sep;125:148–57. doi: 10.1016/j.jclinepi.2020.05.027 (PMC7482905; doi:10.1016/j.jclinepi.2020.05.027)
Supplement: Appendix [file mmc2.docx]

# **APPENDIX**

## **Appendix 1**

| **Medline** (via Ovid) | **Date of search:** 10/12/2018 | | | | **Results =** 232 |
| --- | --- | --- | --- | --- | --- |
| 1. “randomized controlled trial”.pt. 2. (random$ or placebo$ or single blind$ or double blind$ or triple blind$).ti,ab. 3. 1 or 2 4. (animals not humans).sh. 5. ((comment or editorial or meta-analysis or practice-guideline or review or letter) not “randomized controlled trial”).pt. 6. (random sampl$ or random digit$ or random effect$ or random survey or random regression).ti,ab. not “randomized controlled trial”.pt. 7. 4 or 5 or 6 8. 3 not 7 9. (dupilumab or dupixent or benralizumab or faserna or mepolizumab or nucala or omalizumab or xolair or palivizumab or synagis or reslizumab or cingaero).ti,ab,hw,kf,kw. 10. 8 and 9 11. exp dermatitis, atopic/ 12. dermatitis, atopic.ti,ab,hw,kw,kf,sh. 13. eczema, atopic.ti,ab,hw,kw,kf,sh. 14. eczema.ti,ab,hw,kw,kf,sh. 15. atopic eczema.ti,ab,hw,kw,kf,sh. 16. atopic dermatitis.ti,ab,hw,kw,kf,sh. 17. infantile eczema.ti,ab,hw,kw,kf,sh. 18. childhood eczema.ti,ab,hw,kw,kf,sh. 19. neurodermatitis.ti,ab,hw,kw,kf,sh. 20. besniers prurigo.ti,ab,hw,kw,kf,sh. 21. exp asthma/ 22. asthma.ti,ab,hw,kw,kf,sh. 23. asthma/ 24. or/11-23 25. 10 and 24 | | | | | |
|  | |  | |  | |
| **EMBASE** (via Ovid) | **Date of search:** 10/12/2018 | | | | **Results =** 768 |
| 1. (random$ or placebo$ or single blind$ or double blind$ or triple blind$).ti,ab 2. (animal$ not human$).sh,hw. 3. (book or conference paper or editorial or letter or review).pt. not exp randomized controlled trial/ 4. (random sampl$ or random digit$ or random effect$ or random survey or random regression).ti,ab. not exp randomized controlled trial/ 5. 2 or 3 or 4 6. 1 not 5 7. (dupilumab or dupixent or benralizumab or faserna or mepolizumab or nucala or omalizumab or xolair or palivizumab or synagis or reslizumab or cingaero).ti,ab,hw,kw,sh. 8. exp dermatitis, atopic/ 9. dermatitis, atopic.ti,ab,hw,kw,sh. 10. eczema, atopic.ti,ab,hw,kw,sh. 11. eczema.ti,ab,hw,kw,sh. 12. atopic eczema.ti,ab,hw,kw,sh. 13. atopic dermatitis.ti,ab,hw,kw,sh. 14. infantile eczema.ti,ab,hw,kw,sh. 15. childhood eczema.ti,ab,hw,kw,sh. 16. neurodermatitis.ti,ab,hw,kw,sh. 17. besniers prurigo.ti,ab,hw,kw,sh. 18. or/8-17 19. exp asthma/ 20. asthma.ti,ab,hw,kw,sh. 21. Asthma/ 22. or/19-21 23. 18 or 22 24. 6 and 7 and 23 | | | | | |
|  | | | |  | |
| **Cochrane Library** (CENTRAL) | | | **Date of search:** 10/12/2018 | | **Results:** 195 |
| 1. MeSH descriptor: [Asthma] explode all trees 2. MeSH descriptor: [Eczema] explode all trees 3. MeSH descriptor: [Dermatitis, Atopic] explode all trees 4. MeSH descriptor: [Dermatitis] explode all trees 5. MeSH descriptor: [Neurodermatitis] explode all trees 6. #1 or #2 or #3 or #4 or #5 7. (dupilumab OR dupixent OR benralizumab OR faserna OR mepolizumab OR nucala OR omalizumab OR xolair OR palivizumab OR synagis OR reslizumab OR cingaero):ti,ab,kw 8. #6 and #7 | | | | | |

## **Appendix 2**

|  | **Reviewer:** |  |
| --- | --- | --- |
| **General:** | | |
| 1 | Title: |  |
| 2 | First author (surname): |  |
| 3 | Journal: |  |
| 4 | Year of publication: |  |
| 5 | Asthma or eczema trial: |  |
| Design | 6. Non-inferiority trial: |  |
|  | 7. Cluster RCT: |  |
|  | 8. Factorial study: |  |
|  | 9. Crossover study: |  |
| Population | 10. Age range of population: |  |
|  | 11. Severity of disease in population (e.g. mild/moderate/severe) |  |
| 12 | Number of treatment arms: |  |
| 13 | Intervention: |  |
| 14 | Comparator or control: |  |
| 15 | Primary endpoint^†^: |  |
| 16 | Treatment duration (weeks): |  |
| 17 | Number of patients randomised to the study: |  |
|  | | |
| 18 | Has rescue medication been alluded to in the paper? |  |
| 19 | If 18=yes, how did they allude to rescue medication in the paper? |  |
| 20 | Is the protocol available? |  |
| 21 | If 20=yes, how has rescue been defined in the protocol? |  |
| 22 | If 20=yes, how has rescue medication been defined in the protocol? |  |
| 23 | If 20=yes, rescue medication/non-trial treatment allowed: |  |
| 24 | If 23=yes, is the amount of rescue medication used reported in the paper? |  |
| 25 | If 24=yes, how is it summarised – what is the outcome used? |  |
| 26 | If 24=yes, how is it summarised – what is the summary measure? |  |
| 27 | AD/TD/TU/Proportion? |  |
| 28 | If 24=yes, have they summarised rescue medication by arm? |  |
| 29 | If 28=yes, have they tested for a difference between arms? |  |
| 30 | If 29=yes, what is the p-value? |  |
| 31 | If 28=yes, if summarised by arm, what was reported by arm? |  |
| **Primary analysis:** | |  |
| 32 | Have they used a statistical method which takes into account the use of any rescue medication/non-trial treatment in the **primary** analysis? |  |
| 33 | Who is in the primary analysis? (E.g. ITT, mITT) |  |
| 34 | If 32=no, statistical approach to analysis: |  |
| 35 | If 32=yes, statistical approach to analysis: |  |
| **Treatment effect:** | | |
| 36 | Have they used a statistical method which takes into account the use of any rescue medication/non-trial treatment in any **additional** analysis of the primary outcome? |  |
| 37 | Type of treatment effect for primary analysis: |  |
| 38 | What is the treatment effect for the primary analysis: |  |
| 39 | P-value for the TE in the primary analysis: |  |
| **If 32=yes, results of additional analysis of the primary outcome:** | | |
| 40 | If 36=yes, what was the label given to this analysis? (e.g. sensitivity) |  |
| 41 | If 36=yes, what analysis population/approach was used: |  |
| 42 | If 36=yes, type of treatment effect reported in the analysis: |  |
| 43 | If 36=yes, what is the treatment effect in the analysis^¥^: |  |
| 44 | If 36=yes, what is the p-value in the analysis^¥^: |  |
| **Where analysis with and without addressing the use of rescue medication has been undertaken:** | |  |
| 45 | % change of treatment effect: |  |
| 46 | % change in p-value: |  |
| **General:** | | |
| 47 | Software used to implement method: |  |
| 48 | Any other comments: |  |

^†^For the primary endpoint:

1. If only one outcome is listed as being the primary in the final results paper:
   1. Use this
2. If either no outcomes or multiple outcomes are listed as being the primary in the final results paper:
   1. If only one outcome was used in the sample size calculation, use this or,
   2. If no sample size calculated was performed, or a sample size calculation was performed for multiple outcomes, use the first clinical outcome listed in the Objectives/Outcomes section of the final results paper

^¥^ If more than one result presented e.g. if more than one sensitivity analysis was conducted to address use of rescue medication report the largest treatment effect with the smallest p-value.

## **Appendix 3**

| Rescue-adjusted analysis presented | | | | |
| --- | --- | --- | --- | --- |
|  |  | **No** | **Yes** | **Total** |
| P-value for difference between arms | **≤0.05** | 10 | 2 | 12 |
|  | **>0.05** | 8 | 0 | 8 |
|  | **Total** | 18 | 2 | 20 |

**N.B:** The smallest p-value was used in trials with >2 arms

## **Appendix 4**

|  | | Rescue-adjusted analysis | | | Not rescue-adjusted analysis | | |
| --- | --- | --- | --- | --- | --- | --- | --- |
| Study | Active drug | Total n | Odds Ratio †  [95% CI] | Utilized effect size (SE)  [95% CI] | Total n | Odds Ratio †  [95% CI] | Utilized effect size (SE)  [95% CI] |
| (Blauvelt et al., 2017) | Dupilumab Q2W | 421 | 4.46  [2.58, 7.71] | 0.83 (0.15)  [0.52, 1.13] | 421 | 3.42  [2.02, 5.78] | 0.68 (0.15)  [0.39, 0.97] |
|  | Dupilumab QW | 634 | 4.56  [3.00, 7.01] | 0.84 (0.12)  [0.61, 1.08] | 634 | 3.93  [2.66, 5.86] | 0.76 (0.11)  [0.54, 0.98] |
| (de Bruin-Weller et al., 2018) | Dupilumab Q2W | 215 | 3.98  [2.17, 7.32] | 0.76 (0.17)  [0.43, 1.10] | 215 | 3.79  [2.07, 6.94] | 0.74 (0.17)  [0.40, 1.07] |
|  | Dupilumab QW | 218 | 3.43  [1.89, 6.26] | 0.68 (0.17)  [0.35, 1.01] | 218 | 3.25  [1.80, 5.89] | 0.65 (0.17)  [0.32, 0.98] |
| (Simpson et al., 2016) | Dupilumab Q2W | 448 | 5.34  [3.15, 9.30] | 0.93 (0.15)  [0.63, 1.23] | 448 | 4.60  [2.81, 7.65] | 0.84 (0.14)  [0.57, 1.12] |
|  | Dupilumab QW | 447 | 5.18  [3.05, 9.03] | 0.91 (0.15)  [0.62, 1.22] | 447 | 4.14  [2.52, 6.90] | 0.79 (0.14)  [0.51, 1.07] |
| (Simpson et al., 2016) | Dupilumab Q2W | 469 | 6.09  [3.51, 10.90] | 1.00 (0.16)  [0.69, 1.32] | 469 | 5.03  [3.01, 8.58] | 0.89 (0.15)  [0.61, 1.19] |
|  | Dupilumab QW | 475 | 6.18  [3.57, 11.05] | 1.01 (0.16)  [0.70, 1.33] | 475 | 5.19  [3.12, 8.83] | 0.91 (0.15)  [0.63, 1.20] |
| Pooled: all binary studies | | 2444 | **4.79**  **[3.82, 6.02]** |  | 2444 | **4.13**  **[3.34, 5.11]** |  |

Meta-analysis of binary outcome studies

† Active vs. placebo
